# Learning to image and compute with multimode optical fibers

**DOI:** 10.1515/nanoph-2021-0601

**Published:** 2022-01-21

**Authors:** Babak Rahmani, Ilker Oguz, Ugur Tegin, Jih-liang Hsieh, Demetri Psaltis, Christophe Moser

**Affiliations:** Laboratory of Applied Photonics Devices, School of Engineering, Ecole Polytechnique Fédérale de Lausanne, Institute of Electrical and MicroEngineering, Lausanne, 1015, Switzerland; Laboratory of Optics, School of Engineering, Ecole Polytechnique Fédérale de Lausanne, Institute of Electrical and MicroEngineering, Lausanne, 1015, Switzerland

**Keywords:** deep neural network, imaging, multimode fibers, neuromorphic computing

## Abstract

Multimode fibers (MMF) were initially developed to transmit digital information encoded in the time domain. There were few attempts in the late 60s and 70s to transmit analog images through MMF. With the availability of digital spatial modulators, practical image transfer through MMFs has the potential to revolutionize medical endoscopy. Because of the fiber’s ability to transmit multiple spatial modes of light simultaneously, MMFs could, in principle, replace the millimeters-thick bundles of fibers currently used in endoscopes with a single fiber, only a few hundred microns thick. That, in turn, could potentially open up new, less invasive forms of endoscopy to perform high-resolution imaging of tissues out of reach of current conventional endoscopes. Taking endoscopy by its general meaning as *looking into*, we review in this paper novel ways of imaging and transmitting images using a machine learning approach. Additionally, we review recent work on using MMF to perform machine learning tasks. The advantages and disadvantages of using machine learning instead of conventional methods is also discussed. Methods of imaging in scattering media and particularly MMFs involves measuring the phase and amplitude of the electromagnetic wave, coming out of the MMF and using these measurements to infer the relationship between the input and the output of the MMF. Most notable techniques include analog phase conjugation [A. Yariv, “On transmission and recovery of three-dimensional image information in optical waveguides,” *J. Opt. Soc. Am.*, vol. 66, no. 4, pp. 301–306, 1976; A. Gover, C. Lee, and A. Yariv, “Direct transmission of pictorial information in multimode optical fibers,” *J. Opt. Soc. Am.*, vol. 66, no. 4, pp. 306–311, 1976; G. J. Dunning and R. Lind, “Demonstration of image transmission through fibers by optical phase conjugation,” *Opt. Lett.*, vol. 7, no. 11, pp. 558–560, 1982; A. Friesem, U. Levy, and Y. Silberberg, “Parallel transmission of images through single optical fibers,” *Proc. IEEE*, vol. 71, no. 2, pp. 208–221, 1983], digital phase conjugation [I. N. Papadopoulos, S. Farahi, C. Moser, and D. Psaltis, “Focusing and scanning light through a multimode optical fiber using digital phase conjugation,” *Opt. Express*, vol. 20, no. 10, pp. 10583–10590, 2012; I. N. Papadopoulos, S. Farahi, C. Moser, and D. Psaltis, “High-resolution, lensless endoscope based on digital scanning through a multimode optical fiber,” *Biomed. Opt. Express*, vol. 4, no. 2, pp. 260–270, 2013] or the full-wave holographic transmission matrix method. The latter technique, which is the current gold standard, measures both the amplitude and phase of the output patterns corresponding to multiple input patterns to construct a matrix of complex numbers relaying the input to the output [Y. Choi, et al., “Scanner-free and wide-field endoscopic imaging by using a single multimode optical fiber,” *Phys. Rev. Lett.*, vol. 109, no. 20, p. 203901, 2012; A. M. Caravaca-Aguirre, E. Niv, D. B. Conkey, and R. Piestun, “Real-time resilient focusing through a bending multimode fiber,” *Opt. Express*, vol. 21, no. 10, pp. 12881–12887; R. Y. Gu, R. N. Mahalati, and J. M. Kahn, “Design of flexible multi-mode fiber endoscope,” *Opt. Express*, vol. 23, no. 21, pp. 26905–26918, 2015; D. Loterie, S. Farahi, I. Papadopoulos, A. Goy, D. Psaltis, and C. Moser, “Digital confocal microscopy through a multimode fiber,” *Opt. Express*, vol. 23, no. 18, pp. 23845–23858, 2015]. This matrix is then used for imaging of the inputs or projection of desired patterns. Other techniques rely on iteratively optimizing the pixel value of the input image to perform a particular task (such as focusing or displaying an image) [R. Di Leonardo and S. Bianchi, “Hologram transmission through multi-mode optical fibers,” *Opt. Express*, vol. 19, no. 1, pp. 247–254, 2011; T. Čižmár and K. Dholakia, “Shaping the light transmission through a multimode optical fibre: complex transformation analysis and applications in biophotonics,” *Opt. Express*, vol. 19, no. 20, pp. 18871–18884, 2011; T. Čižmár and K. Dholakia, “Exploiting multimode waveguides for pure fibre-based imaging,” *Nat. Commun.*, vol. 3, no. 1, pp. 1–9, 2012; S. Bianchi and R. Di Leonardo, “A multi-mode fiber probe for holographic micromanipulation and microscopy,” *Lab Chip*, vol. 12, no. 3, pp. 635–639, 2012; E. R. Andresen, G. Bouwmans, S. Monneret, and H. Rigneault, “Toward endoscopes with no distal optics: video-rate scanning microscopy through a fiber bundle,” *Opt. Lett.*, vol. 38, no. 5, pp. 609–611, 2013].

The dependence of the aforementioned methods [1–15] on the phase measurement is also their weakness. This is rooted in two reasons. First is the necessity of having a nontrivial phase measurement apparatus. A holographic experiment requires an external reference beam brought to the output of the fiber to generate an interference pattern from which the complex optical field (amplitude and phase) can be extracted. Although some work has shown that the reference beam can also be sent through the same MMF [16], multiple quadrature phase measurements must be done to extract the phase.

The second reason is the sensitivity of the phase to external perturbations. Any mechanical variation or thermal variability, among others, could drift the phase of the reference wave. Upon significant change of the phase, the calibration process needs to be repeated. Therefore, careful phase tracking needs to be implemented to correct for phase drift, which further complicates the implementation.

Thereby, a method that can characterize the MMF without using the phase information of the output wave while at the same time is as general as the gold standard methods is highly desired. Recently, data driven methods have been applied for characterizing scattering media and MMFs. These techniques rely on inferring the statistical characteristics of light propagation through the MMF system through examples. Some works have used convex optimization to infer the transmission matrix from intensity measurement [17, 18]. Others use Bayesian inference to infer the input of the system. For the latter methods, an estimate of the TM of the system is first approximated using some probe signals sent through the medium. Afterwards, an additional step based on Bayesian inference is taken to infer the system’s input either for imaging [19], for projection [20] or computation [21]. Although these works are promising steps for phase-independent characterization of the MMF, they either lack generalization or require independent computation each time a pattern needs to be imaged or projected. For example only limited types of images, mostly sparse such as spots, was projected with these methods [22]. A data-driven method that is able to generalize to all level of image complexities, one that can learn input and output relation end-to-end while all noise sources are present (even when the noise is time-dependent) and one that does not require additional computation for every pattern is more advantageous.

In what follows, we review recent works that use modern data-driven deep neural networks (DNNs)-based methods for imaging, projection in scattering media and specifically MMFs. We show that these methods considerably simplify the measurement system and experiments and show that they can correct external perturbations as well. We also show recent works that MMF can be used as a medium to do optical computing [23], [24], [25], [26], [27], [28], [29], [30], [31], [32].

## 1 Learning to image

**Figure 1:**
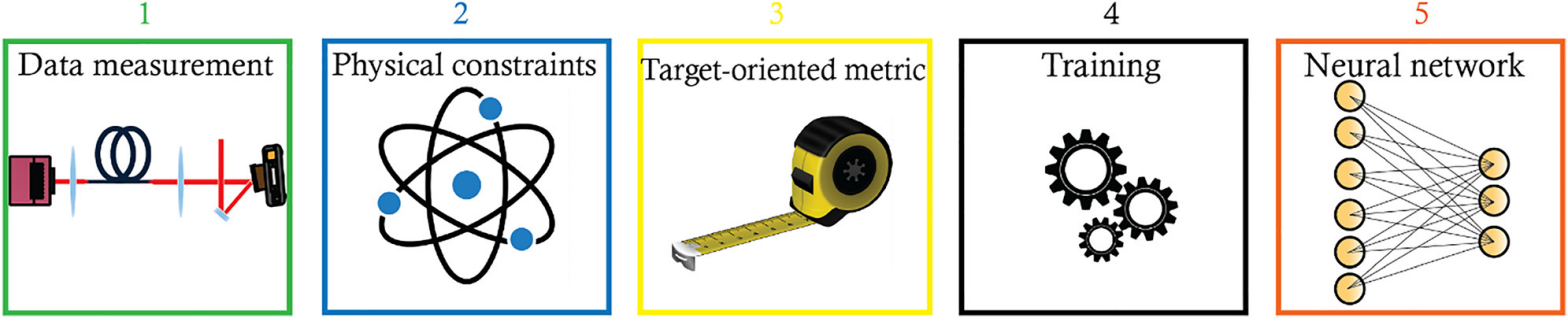
General framework for learning of physical systems such as the system of an MMF.

Learning-based methods for imaging through scattering media and particularly MMFs seek to retrieve the input information (usually a 2D image) entering the system from intensity-only measurements of the output. In particular, as the phase information of the wave exiting the distal facet of the fiber is lost due to the squared-law of the detector (a CCD or CMOS camera), these methods seek to reconstruct the input from statistical characteristics of the system learned from data. It should be noted that such a problem is highly ill-posed as many inputs can result in the same amplitude profile at the output of the fiber that only differ in their respective phase information. A general framework for learning physical systems, such as that of the MMFs, depicted in Figure 1 involves acquiring many samples of the system’s input–output (1) which is governed by physical propagation (2). A quantifiable metric is then chosen for the inverse problem (3) from which a neural network is trained (4). Finally, the trained network is used for inference i.e. for generating the system’s output from an input.

In particular, examples of the input–output obtained from the system are used to learn the backward mapping of the system without estimating the forward mapping. The learning is carried out by minimizing a loss function constructed in the following way:

(1) $$\hat{\theta }=\underset{\theta }{\text{argmin}}\left[M\left(x,A_{\theta }\left(y\right)\right)+\alpha \left|\right|\theta |{|}_{l_{p}}^{2}\right]$$

where x and y are the input and output of the system. We note that x in general is complex, whereas y is always a positive real number. $\hat{x}$ is the solution of the this optimization problem $\hat{x}=A_{\hat{\theta }}\left(y\right)$ . The operator M represents a metric between the predicted output of the network $A_{\theta }$ that is parametrize by θ . Often time, regularizing θ provides better reconstruction fidelities. Therefore, a regularizing term ( $l_{p}$ norm) is added to the loss function that could be tuned by the hyperparameter α . The regularization term constraints the optimization to look for a sub-space (instead of the full space of all-possible θ ) that the solution is expected to exists in. Reference [33] provides an overview of the necessity of regularization for inverse problems. The loss function is minimized by taking gradients with respect to the learnable parameters of the mapping function A , i.e. θ , in a process known as gradient descent. Once converged, the mapping function is an estimator of the backward mapping of the MMF system that predicts the input patterns of the system from the corresponding outputs. Depending on the modulation type of the input beam, whether the information is incorporated in the amplitude, phase or both of the formers, *x* could be real or complex. It is expected that reconstruction in the case of complex or phase modulation be more difficult than the amplitude modulation as in the former cases, either the real and imaginary parts of the input pattern (complex modulation), two dependent unknowns, or its logarithm (phase modulation), hence a nonlinear function, is to be reconstructed.

A great number of recent works studied the use of data-driven methods for computational imaging in scattering media [34], [35], [36], [37], [38], [39], [40] for example for microscopy [41], [42], [43] and imaging under low-photon condition [40]. A review on these methods is studied here [44].

**Figure 2:**
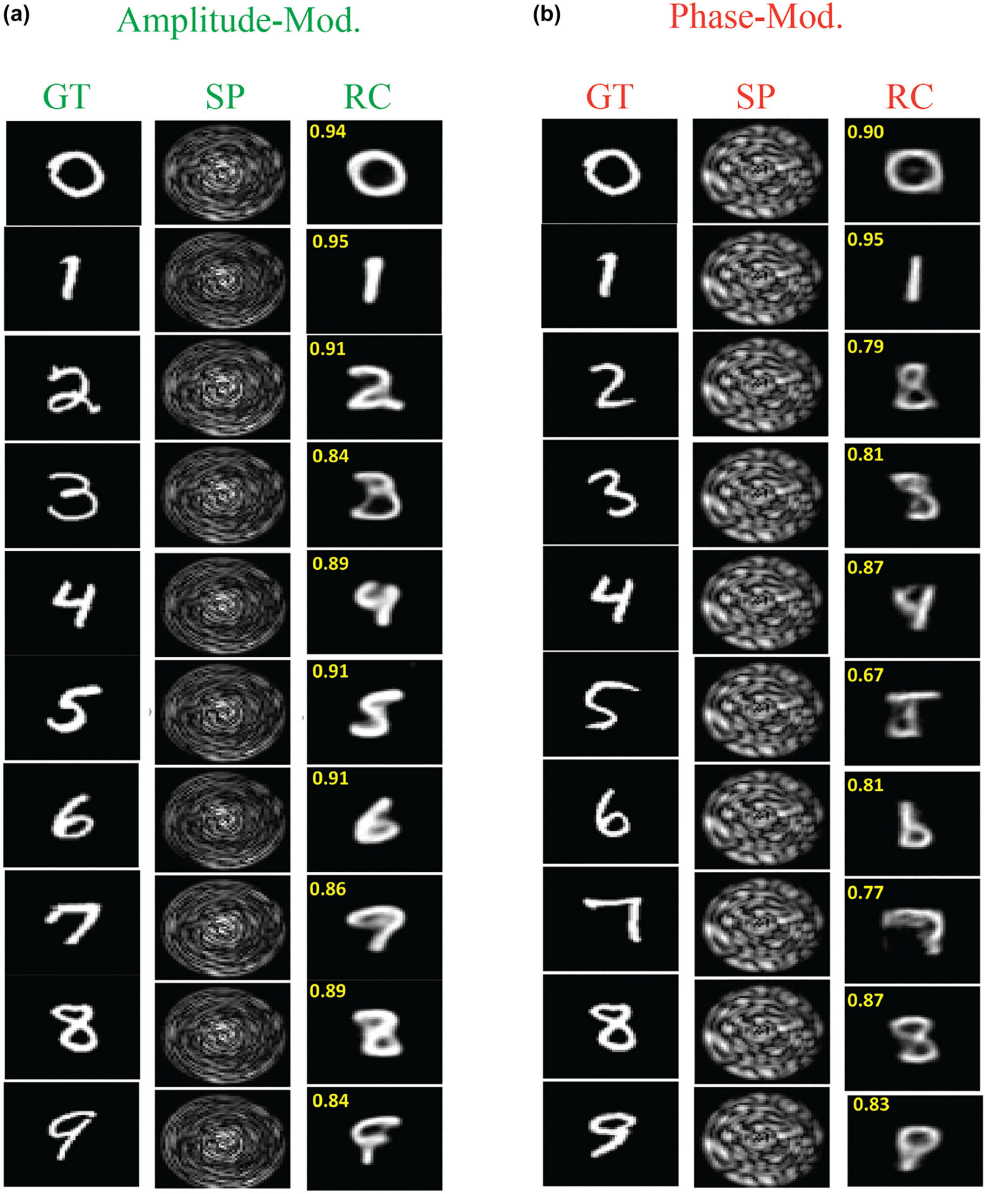
Performance of the network in transfer learning the reconstruction of the input amplitudes/phases from the output amplitude speckle patterns when the DNN is trained with the handwritten Latin alphabet. The speckle pattern for each image is obtained using the TM of the system. (a) Reconstructed amplitude input patterns and (b) reconstructed phase input patterns of digit images. The performance metric (2-dimensional Pearson correlation between the GT images and RC images) are shown as inset. Figures adopted from [45].

In MMFs, using a convolutional network, Rahmani et al. [45] attempted to reconstruct the input information scrambled upon propagation through a 0.75 m piece of step index 50 μm diameter silica core size MMF. Both types of the modulation, i.e. phase or amplitude, were tested. Examples of the reconstructed inputs for both modulations are plotted in Figure 2. After training, the same network could be used to reconstruct images from a different class. Although the authors show that their network could transfer its knowledge for retrieval of other types of images, the retrieval performance deteriorates when the images become significantly different with the training set.

**Figure 3:**
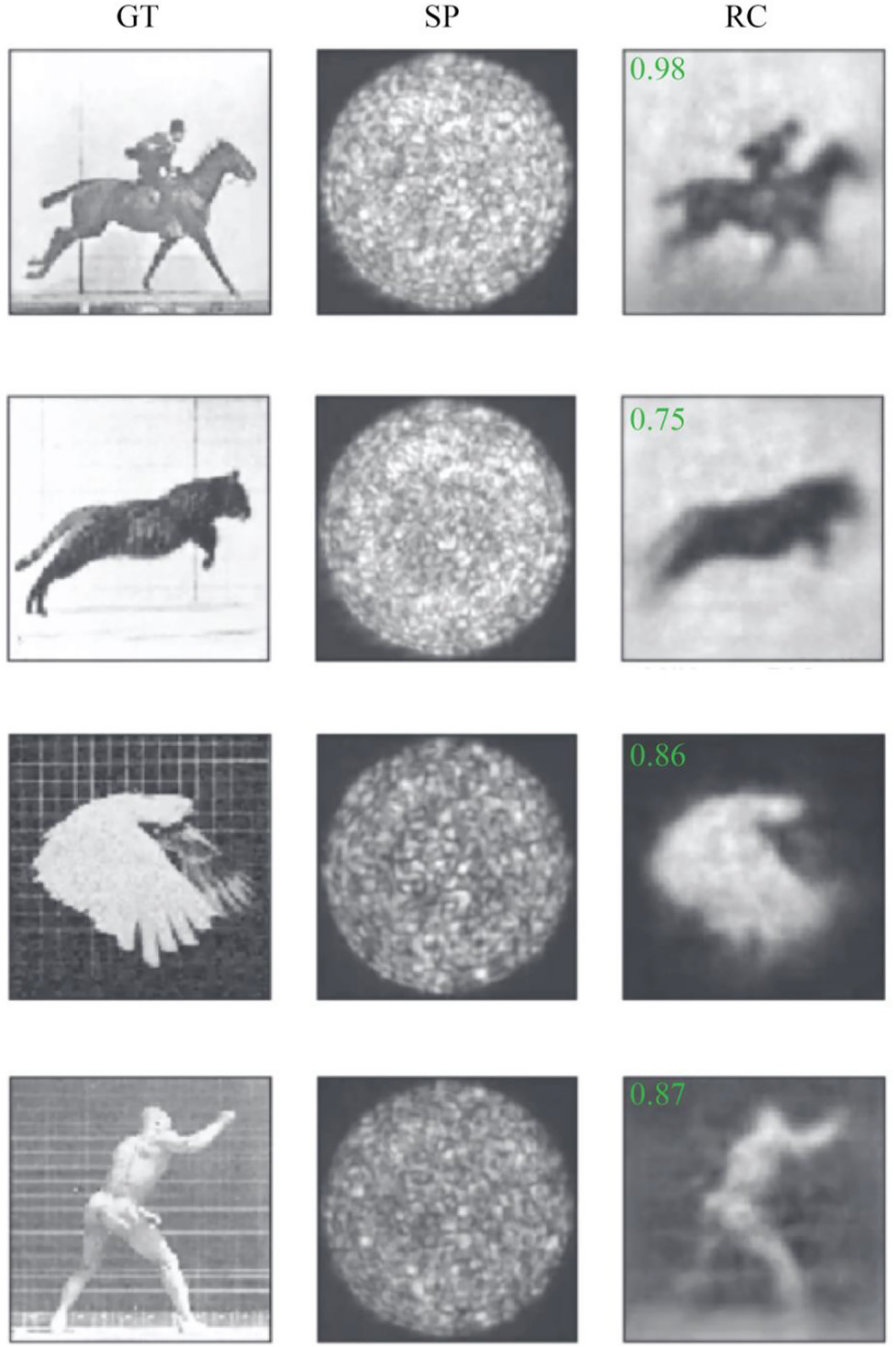
Performance of the network in reconstruction of the input amplitude from the output amplitude speckle patterns when the DNN is trained with natural images. The figure is adopted from [46] with modifications. The performance metric (2-dimensional Pearson correlation between the GT images and RC images) are shown as inset.

To overcome this problem authors in [46] use a combination of two networks for reconstructing the input of the fiber system. In particular, first a network retrieves back the input pattern of the fiber from intensity-measurements of the outputs. The predicted input is then fed to a second network that maps this inferred input pattern to the original scrambled output initially given to the first network. In essence, the first network is learning the backward mapping of the fiber from intensity-measurements to the modulator pattern while the second network is learning the forward mapping. Example of images reconstructed via this double network is shown in Figure 3. The training dataset used here are from natural images (images of everyday objects). As evident, these images have higher structural complexity as compared with the sparse-like images used in the work of Rahmani et al. [45].

Both of the works of Caramazza et al. and Rahmani et al. [45] impose a computational expensive burden due to their network architecture of the training algorithm. Authors in [47] proposes a simpler network for reconstruction of the images scrambled through MMF. They use a fully-connected network to reconstruct sparse-like images of digits and obtain the same level of fidelity as compared with the previous works.

In another work [48], Borhani et al. uses neural networks for classification as well as reconstruction of the inputs of an MMF system for various lengths of the fiber starting from 0.1 m up to 1 km. Both amplitude- and phase-modulated SLM patterns are tested. It was shown that, for fibers up to 10 m in length, phase-modulated input provides slightly better classification accuracies, probably due to the more uniform distribution of the injected light across the fiber modes. For amplitude-modulated inputs, there is a selective spatial excitation of the fiber modes at the input facet, which may limit the number of modes that actually participate in transporting the information. On the other hand, for the 1 km fiber, the amplitude-modulated proximal input image provides better classification accuracies. Authors attributed this to distribution of the information among all modes along the long propagation distance of the fiber. Additionally, despite the pronounced sensitivity of the fiber to external perturbation specifically in the case of the long 1 km length, it was observed that the network learns to correct for the perturbation and successfully reconstruct the information.

In keeping with the results of Borhani et al. [48], authors in [49] attempted to learn perturbed system of MMFs. In particular, the fiber was moved around a number of different but fixed configurations where in each position examples of the input-output of the system were collected. A network was trained with the entire dataset (combined dataset from multiple configurations) to reconstruct the input of the system. Even though the fiber undergoes severe change from one configuration to the other, it was shown that reconstruction of the input is still possible. This might be an advantage of learning-based methods as compared to their non-learning counterparts; for in the latter case, when the system is moved (unless for small lengths of MMFs [50] – few hundreds of millimeter- or some specialty MMFs such graded-index (GRIN) [51] or multicore), a recalibration of the entire system is necessary. To correct for perturbations, we note that learning-based methods require samples from different perturbed configurations while the non-learning method require additional characterization process.

Similarly in [52], authors applied deep learning to the image retrieval problem that shows robustness to fiber deformations as large as few millimeters. By drawing from a method that combines data from different configurations of the MMF (configuration learning), images decorrelated by a factor of 10 (Pearson correlation of 0.1) because of fiber bends, were reconstructed with high fidelities. The authors attribute this success to DNNs learning invariant properties in the speckle produced for different fiber conformations. Similar methods have been applied to more drastic fiber perturbations (smaller bent radius), for example in [53] where authors show successful reconstruction of the input image for 5 cm fiber bend.

**Figure 4:**
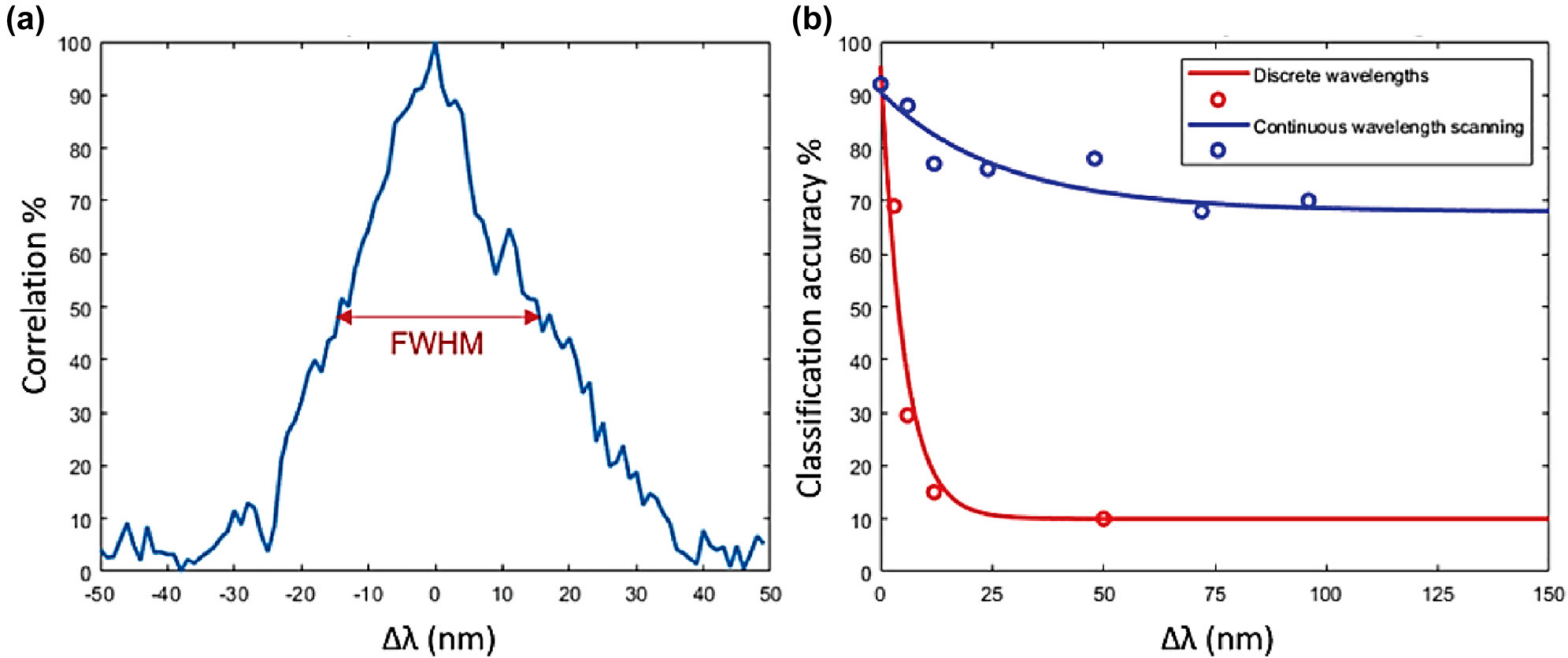
Speckle intensity correlation as a function of wavelength drift bandwidth; (b) Classification accuracy for datasets collected for the two cases discussed in the text: DNN trained at 800 nm wavelength and tested at data collected at different wavelengths (red circles) and DNN trained with data that include the perturbation (blue circles). Figures adopted from [54].

Another source of perturbation is the drift in the wavelength of the laser source that decorrelates the output intensity with time. In a study conducted by Kakkava et al. [54], [55], [56], it was shown that the DNNs can correct for the decorrelation rendered by the wavelength change of the fiber with an extended bandwidth. A classification of the output intensities into single digit numbers was conducted using a multimode GRIN fiber of length 10 cm and core size 62.5 um diameter at central wavelength 800 nm. Figure 4(a) plots the deterioration of the speckle pattern versus wavelength changes. As evident, at a half width bandwidth of 50 nm, the output pattern gets entirely decorrelated. Two experiments where conducted. In one, a network is trained with examples of input-output, only at the central wavelength and then was tested on examples at other wavelengths while in the second experiment, the network was trained with examples from multiple wavelength points and tested on various wavelengths within the range of wavelengths used for training. As evident in Figure 4(b), the performance of the network is substantially higher in the latter experiment.

External perturbations that are detrimental for imaging could be harnessed for sensing in MMFs. Authors in [57] use deep learning for sensing, such as temperature for example, using the complex optical interference output of an MMF. The method is shown to work even when the information is buried in strong undesired noise. In other line of works, the spectral output of the MMF is used for sensing mechanical perturbations such as bends [58, 59] along the fiber.

## 2 Learning to project

**Figure 5:**
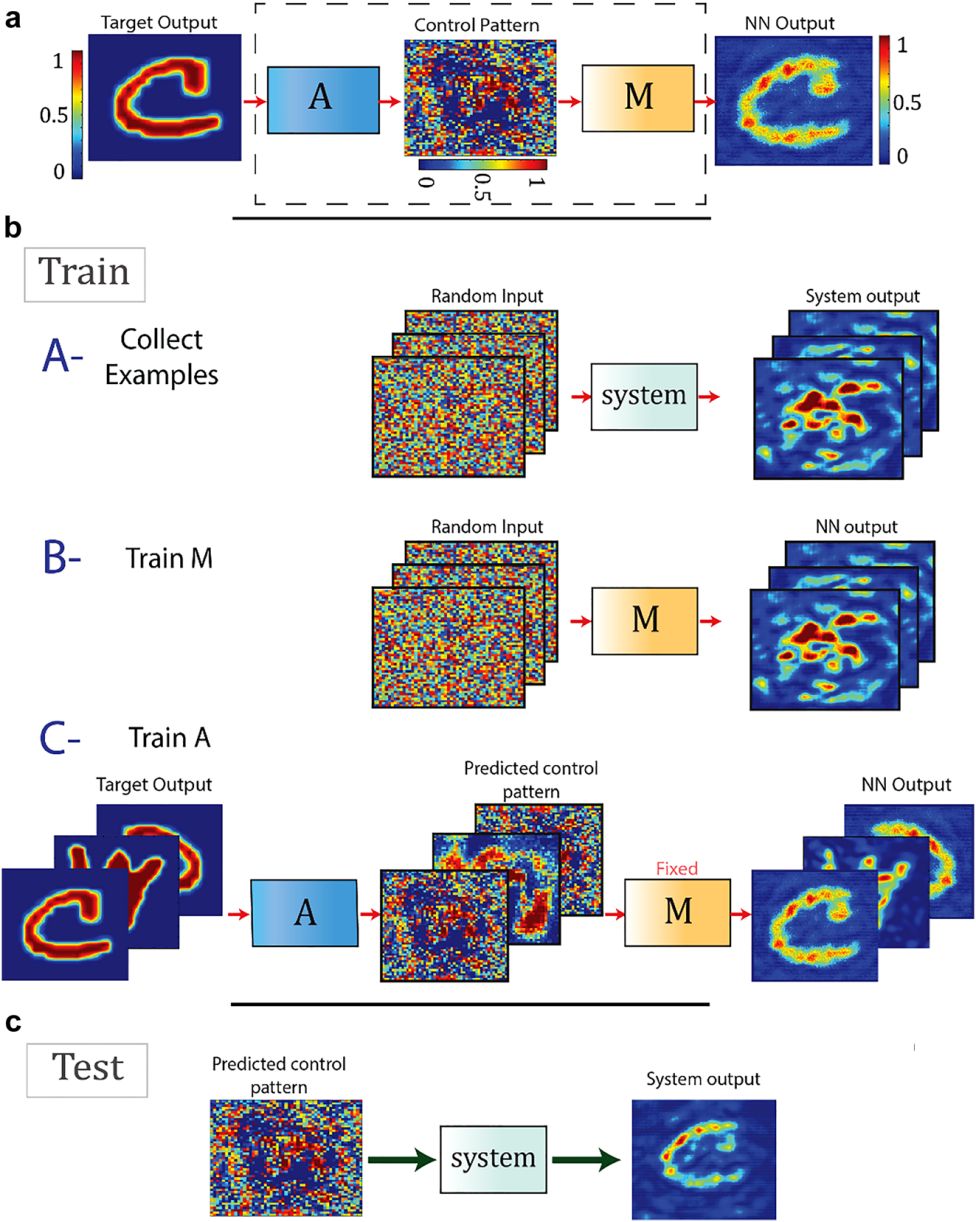
The projector network consists of two subnetworks: the model (M) and the actor (A). Once trained, the subnetwork actor accepts a target pattern desired to be projected at the output MMF to generate an SLM pattern. The training procedure is carried out in three steps. (i) A number of input control patterns are sent through the system and the corresponding outputs are captured on the camera. (ii) The subnetwork model is trained on these images to learn the mapping from the SLM to camera, so the model is essentially learning the optical forward path of light starting from its reflection from the SLM, propagation through the MMF and finally impinging on the camera. (iii) While the sub-network model is being fixed, the actor is fed with a target image and is asked to produce an SLM image corresponding to that target image. The actor-produced SLM image is then passed to the fixed subnetwork model. (c) The test procedure is carried out by feeding the target image to the trained subnetwork actor and acquiring the appropriate SLM image corresponding to that target image and sending it through the system. Figure adopted from [60].

**Figure 6:**
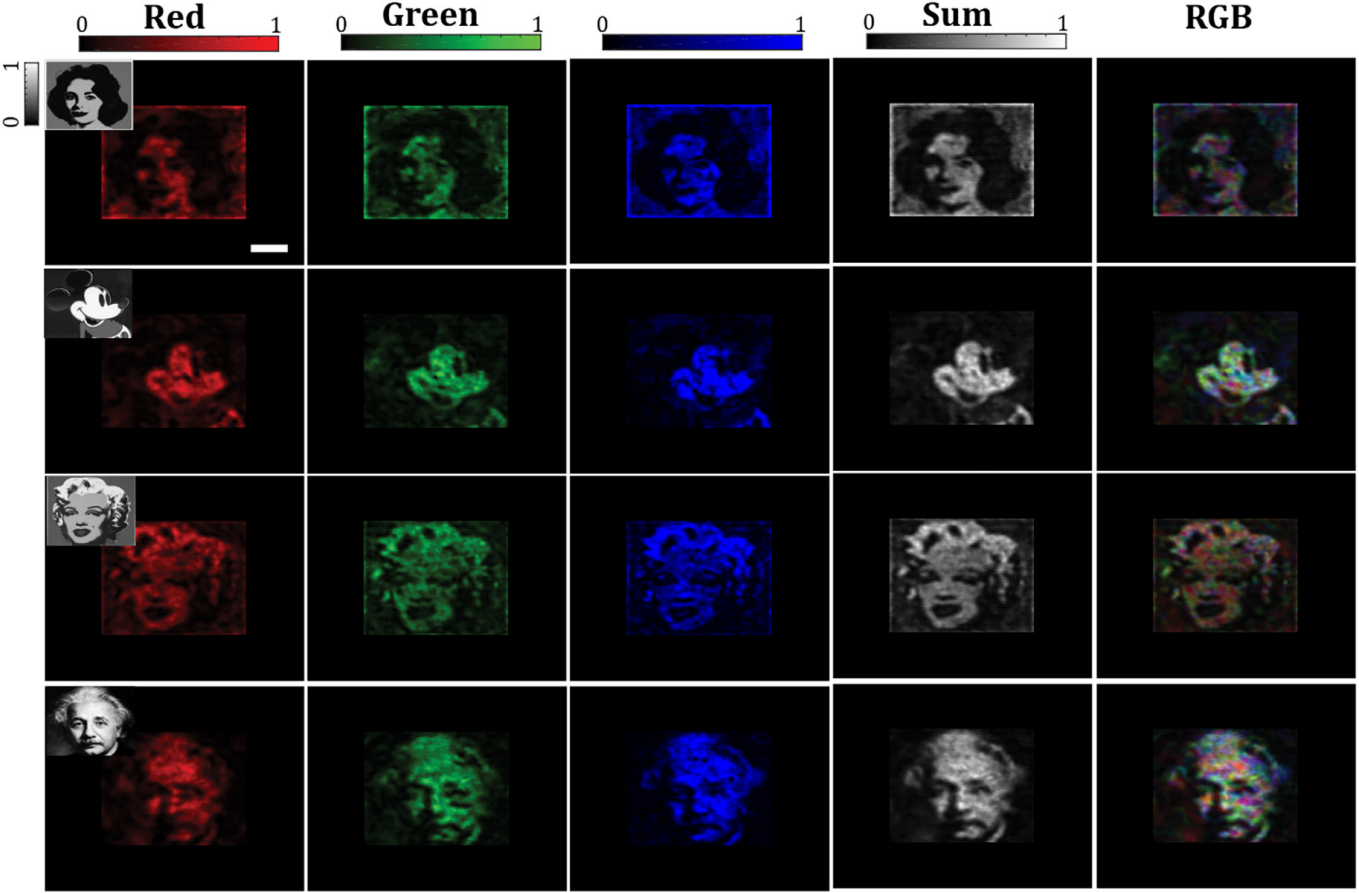
Continuous grey-scale image projection. Examples of natural-scene continuous grey-scale target and experimentally projected images being sent through the MMF and captured on the camera for colors red, green, blue and the three-channel RGB as well as the superposition of all three colors in one channel (sum) are shown. Figure adopted from [60].

Learning-based methods for projection through scattering media and MMFs [60, 61] seek to find the correct input pattern that upon propagation through the fiber produces a desired image at its distal side. It is again assumed that the fiber system is characterized without resorting to holographic measurements. In other words, the phase information of the wave exiting the fiber is not measured and only intensity measurements of the output are available. A similar learning of the MMF system as in the imaging case could not be carried out here since *a priori*, no proper examples of the correct input for producing a desired output image is available. In [60] authors propose to use a combination of two networks as a solution to this problem. In particular, one network, referred therein as model network, is trained to mimic the forward propagation path of the MMF from where the incoming wave is modulated by an SLM until it reaches the camera. A second network, referred to as Actor, is then trained to obtain the inverse of the forward path. These two networks are trained one at a time after each other so as to the actor network uses an estimate of the forward propagation of the MMF learned by the model to find the correct input required for projection of the desired target output. The handshake between the two-network method and the MMF system (achieved through updating the model with the experimental data received from the MMF) act as a feedback which allows the actor to move toward the appropriate distribution of SLM patterns that is required for producing the desired target outputs. A sketch of the training procedure for this network is depicted in Figure 5. Results of the projection through the MMF are shown in Figure 6. Authors in [60] compared the projection fidelity of the two-network method with that of the holographic-based TM approach for various dataset shown in Table 1.

**Table 1:** Neural network and TM image projection average fidelities (in percent) for various dataset. Data adopted from [60].

Dataset (1000 samples)	Average variance			
	NN	TM	NN	TM
Latin alphabet	92.4	96.9	3.7	0.8
Digits	92.5	97.1	3.5	0.7
Random sketches	83.9	90.3	3.9	1.2

## 3 Learning to compute

In addition to using digital computers to image and project with MM optical fibers, they can also be used to perform computational tasks since a complex transformation occurs as light propagates through a fiber due to linear and nonlinear effects. One of the main transform mechanisms in multimode fibers is linear scattering and this phenomenon was already utilized for computational processes in the context of complex media. Using TiO_2_ nanoparticles on a microscopy slide as the scattering medium, Saade et al. [23] showed that linear scattering process could be used as a random projection kernel on the data and can improve the accuracy on classification tasks. In addition, this improvement was further analyzed by Gao et al. [24], using the combination of a simple linear classification algorithm and speckle formation by a scattering medium from input images at different wavelengths, they demonstrated that this combination can perform comparably with a deep convolutional neural network trained directly on input images. Moreover, in the study by Dong et al. [25], linear scattering process provided the random projection step as a part of recursive computation scheme, realizing optical reservoir computing. Nonlinear activation function on this recurrent neural network was modulus square operator, since the final state of electromagnetic field could be measured as intensity on a camera sensor.

Sunada et al. [26] demonstrated the transform of information thanks to the dynamics of speckle formation inside MMFs. The information is encoded as the phase of spatially single-mode input laser, and the intensity of the speckle pattern at different locations of the output beam was recorded. This transform of data is then shown to be applicable to the task of chaotic time series prediction. Similarly, Paudel et al. [27] encodes the information with a spatial light modulator to a beam which is then propagated inside an MMF. The output speckle is then recorded with a camera. This output pattern is then fed back along a new input pattern at the input of the MMF. Nonlinearity is introduced at the coding level, from phase to intensity and from the field amplitude to intensity conversion at the camera. In another study, the crosstalk between cores and the spatial control of gain inside a multi-core active fiber is proposed to be used for realizing an optical computer that operates as a neural network [28].

Even though optoelectronic interfaces such as cameras provide nonlinearity naturally, to implement a fully optical neural network, or to simply benefit from optical domain’s advantages such as parallelism, many groups aimed to achieve nonlinearity optically with different approaches. Using relatively mature silicon photonics technology, Jha et al. [29] built a cavity-loaded Mach–Zehnder interferometer device that can perform nonlinear activation functions. This device could implement different nonlinearity functions and achieve generalization in different tasks. Miscuglio et al. [30] used a quantum dot in conjunction with gold nanoparticles to achieve all-optical nonlinearity such as saturable absorption. In the study by Zuo et al. [31], the transitions between states of laser-cooled atoms were tuned to achieve all-optical nonlinearity. Then, these nonlinearities were placed in a free-space optical setup to be the activation function inside an optical neural network and this network could classify order and disorder phases of a statistical Ising model.

**Figure 7:**
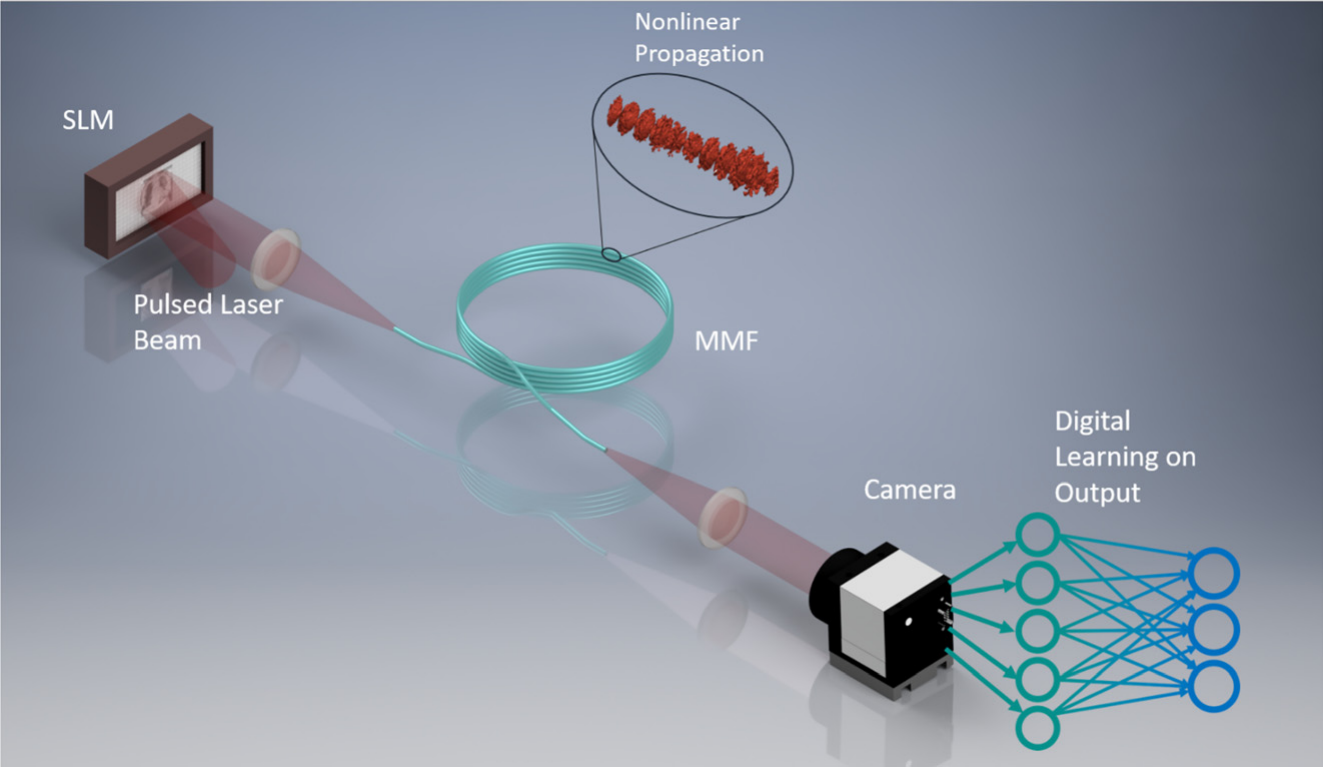
A simplified schematic of the nonlinear optical data transformation performed by an MMF. The change of spatial distribution of the electromagnetic wave with nonlinear propagation is shown in the inset and the training of a single digital neural network layer on the collected data is illustrated next to the camera. Figure illustrating the system of [32].

Instead of using additional optical components or relying on electro-optic devices for nonlinearity in the data transformation, which is indispensable for successful generalization in many computational tasks, Teğin et al. [32] demonstrated that optical nonlinearities inside an MMF can be effectively utilized for the same purpose, and it improves the performance on different machine learning tasks. In this study, an optical system consisting of an input SLM, coupling optics from the SLM to the fiber, the MMF, and a 2D camera that records the spatial distribution of the light intensity of the field emerging from the far end of the fiber, carries out a transformation of the data that modulates the input SLM to the detector distribution as shown on Figure 7. This transformation is generally nonlinear due to square-law detection. In addition, when the light intensity of the light source is increased sufficiently, optical propagation inside the MMF becomes nonlinear due to the nonlinear response of the material in the MMF. This transformation becomes a useful computational element when the fixed operation performed by the MMF is complemented with electronic adaptive elements that can be trained to produce a favorable operation for the combined system. This is in some ways similar to the combination of a DNN with an MMF to perform imaging as described earlier. The main difference between the computational and the imaging cases is the importance of optical nonlinearity in the MMF. In what follows, we describe recent experimental results demonstrating MMF-based optical neural networks. The key advantage of this optical implementation is the power efficiency compared to digital techniques.

During the optical transformation of the data from SLM to the camera, the encoded information can be considered to be decomposed into the modes of the MMF each having a magnitude and phase value. Thanks to the fixed linear and power-dependent nonlinear optical effects, these values change, and energy is coupled from one channel of the fiber to another one. This high-dimensional and nonlinear transformation works as the engine of the computation mechanism and in the following results, the generalization performance of this transform is presented when it is combined with a simple linear regression step after the data is recorded on a digital computer. The strength of nonlinear mixing is power dependent. The reported experiments were done at the optimum power level which was obtained experimentally by changing the optical power. This power level corresponds to the case where nonlinearity is significantly effective but slightly weaker than it would be necessary to create Kerr induced beam cleaning, which causes an all-to-one mapping and decreases generalization performance.

Table 2 lists the classification performances of the MMF computation system on several databases, these illustrate that the complex transformation of the data yields generalization on a diverse set of problems.

**Table 2:** The performance metrics of the system presented in [32], on different classification and regression tasks.

Dataset	Abalone	Age prediction from face	Audio digit – digit recognition	Audio digit – speaker recognition	COVID-19 x-ray
Normalized mean squared error	0.126	0.167	—	—	—
Test set classification accuracy	—	—	94.5%	95.2%	83.2%

## 4 Discussion and outlook

A variety of examples illustrate the power of DNNs applied to MMFs for imaging and projection which relies only on a simple intensity detection at the MMF’s output. Particularly, it was shown that DNNs can learn to reconstruct the input entering an MMF from output speckles. Several classes of input images were reconstructed, ranging from sparse-like images such as MNIST to more complex natural looking pictures. The fidelity of reconstruction significantly depends on the type of images that the DNN is trained with. Importantly, a DNN trained on sparse images cannot be used for reconstruction of natural images. The same is true for the projection in which the DNN is task with finding the correct input required for projection of a desired image at the output of the MMF.

Moreover, DNNs were used to correct perturbations imposed on the MMF system. It was shown that a DNN can correct for wavelength and positional drifts with acceptable fidelities as long as the drift is within the range for which training data were available. In other words, the performance decreases when the DNN is expected to extrapolate.

The strong dependence of the DNN’s performance on the availability of training data could limit its applicability especially when obtaining data is expensive, for example in microscopy. One possible line of research is using techniques such as few-shot learning [62] in which a DNN is trained with few training examples.

In addition to imaging and projection, MMFs are also shown to efficiently perform useful data transformations, when the input data is encoded either temporally or spatially. Similarly, the random mapping occurring inside MMFs were also utilized with other complex media, and high performances were achieved with the help of nonlinearity on optoelectronic interfaces. However, optoelectronic conversions could be the main limiting factor in different computing architectures for considerations of energy consumption or speed. On the other hand, nonlinear activation functions play a crucial role in the generalization ability of neural networks, hence a growing body of work investigated the possible implementations of all-optical nonlinear activation functions. Silicon photonics, plasmonics or material response based optical nonlinearities were shown to be suitable for the role of activation functions in the context of neural networks. However, performing this operation with spatially multimode beams in a physically compact structure was only possible with multimode fibers. The spatiotemporal nonlinear optical effects in MMFs were shown to perform a nonlinear mapping that improves the performance in machine learning tasks significantly. This is especially noteworthy, because for decades the nonlinearities inside optical fibers were regarded as disruptive effects and many approaches were developed to compensate for them. However, under the light of the recent results, these effects hold the promise of naturally improving performances in information processing tasks. The integration of information transmission and transformation together inside optical fibers could decrease the strain on silicon electronics based digital computing systems, which are now reaching their limits in terms of miniaturization and parallelization.
